# Development and Validation of the Turkish Spiritual Amnesia Scale (TSAS): Measuring Spiritual Disengagement Among Youth

**DOI:** 10.1007/s10943-025-02370-y

**Published:** 2025-07-04

**Authors:** Nesrullah Okan, Nihat Köse, Yusuf Celal Erol, Mine Sayin-Kiliç

**Affiliations:** 1 Department of Educational Sciences, Guidance and Psychological Counseling, Fırat University, Elazığ, Turkey; 2 Department of Educational Sciences, Department of Education Management, Fırat University, Elazığ, Turkey; 3 Department of Educational Sciences, Guidance and Psychological Counseling, Bartın University, Bartın, Turkey

**Keywords:** Spiritual amnesia, Youth, Scale development, Spirituality, Cultural context, Turkey, Psychometrics

## Abstract

The present study introduces the Turkish Spiritual Amnesia Scale (TSAS), a recently developed psychometric instrument intended to assess spiritual disengagement among Turkish youth. The theoretical framework underpinning the TSAS is predicated on the conceptualisation of spiritual amnesia as the progressive weakening of cognitive, emotional, and behavioural ties to spiritual beliefs and practices. This phenomenon is frequently precipitated by modernisation, digitalisation, and individualisation. The scale development process was guided by theoretical foundations such as Pargament’s spiritual coping theory and Fowler’s faith development theory. The process involved item generation, expert review, pilot testing, exploratory and confirmatory factor analyses, and reliability assessments. A total of 296 emerging adults participated in the EFA and 242 in the CFA. Additionally, criterion validity was assessed using a separate sample of 54 individuals. The findings obtained supported a unidimensional structure that explained 61.17% of the variance, with acceptable model fit indices (CFI = 0.911, RMSEA = 0.08). The TSAS demonstrated excellent internal consistency (Cronbach’s *α* = 0.964) and robust test–retest reliability (*r* = 0.84, *p* < 0.001). The criterion validity of the scale was confirmed through a positive correlation with the Spiritual Contradiction Scale (*r* = 0.448, *p* < 0.001). The TSAS is a culturally grounded, theoretically sound, and psychometrically reliable instrument for the assessment of spiritual disengagement. Its application in psychological, spiritual, and sociocultural research has the potential to contribute to understanding contemporary forms of spiritual detachment in secularising contexts. Furthermore, the scale offers practical value for professionals working in youth counselling, spiritual assessment, and mental health services.

## Introduction

### Importance and Scope of the Study

In recent years, the weakening of spiritual bonds and lack of interest in spiritual values among young individuals have been the subject of increasing academic interest in the fields of psychology, religion and health (Smith & Snell, 2009; King & Boyatzis, 2015). This condition, which can be called “spiritual amnesia”, is defined as the weakening of the individual’s connection with his/her religious and spiritual identity over time, decreasing interest in religious practices and distancing from meaning structures related to spirituality (Pargament, 1997; Pargament et al., 2013a, 2013b).

The main social dynamics affecting this process are modernisation, digitalisation, and individualisation (Campbell, 2012; Turner, 2010). Modernisation reduces the influence of traditional religious authorities and leads to the prominence of individual interpretations in accessing religious knowledge (Inglehart, 2020). Especially in Turkey, the decline in trust in religious leadership (Çınar, 2018) and transformations in the family structure are important factors that trigger spiritual detachment. Recent studies have also explored the link between spiritual functioning and maladaptive behavioural patterns, suggesting that disruptions in spiritual orientation may contribute to higher vulnerability to violence or psychological distress (Ekşi & Okan, 2021).

Digitalisation, on the other hand, both facilitates access to religious knowledge and brings about a move away from traditional practices (Smith & Snell, 2009). The concept of “digital amnesia” suggests that individuals’ over-reliance on digital information negatively affects not only cognitive memory but also spiritual memory (Robert et al., 2024; Kanbay et al., 2025).

Studies in the field of memory and amnesia point out that forgetting is not only a biological but also a socio-semantic phenomenon (Ketonis et al., 2025; Zhang & Andl, 2022). Continuous access to information, especially in the digital environment, may cause individuals to have difficulty in keeping spiritual content in their minds (Kartsonaki et al., 2022; Natale et al., 2024). Research has shown that spiritual meaning-making plays a critical role in trauma recovery, and its disruption may hinder psychological resilience (Kızılgeçit et al., 2024).

Recent studies conducted in Turkey show that young people’s ties with traditional religious structures are dissolving and the tendency towards individual spiritual pursuits is increasing. This tendency, combined with secularisation through social media and digital culture, has evolved into a ground where religious knowledge and practice are reproduced in a superficial manner.

The aim of this study is to scientifically measure the level of spiritual disconnection of individuals through the “Turkish Spiritual Amnesia Scale” (TSAS) developed specifically for Turkish youth and to contribute to the literature.

### Concept of Spiritual Amnesia and Research Problem

Spiritual amnesia is a contemporary concept defined as the individual’s loss of connection with his/her religious and spiritual identity, decreased interest in related values, and distancing from belief systems (Hill & Pargament, 2003; Pargament, 1997). In the literature, this phenomenon is associated with concepts such as “spiritual disengagement”, “spiritual forgetfulness”, and “faith detachment” and has effects on the individual’s search for meaning, psychological resilience, and social belonging (Smith and Snell, 2009; King & Boyatzis, 2015).

The process of spiritual amnesia consists of cognitive (questioning or forgetting belief), emotional (loss of inner peace), and behavioural (decreased participation in rituals) dimensions (Kanbay et al., 2025; Okan & Ekşi, 2025).

Sociocultural factors such as modernisation, digitalisation, and individualisation accelerate this process (Çınar, 2018; Inglehart, 2020). Digital amnesia, which emerges with the effect of digitalisation, can make it difficult to keep spiritual content in mind (Anderson & Subbulakshmi, 2024; Robert et al., 2024).

Although studies conducted in Turkey point to the existence of scales developed in areas such as spiritual resilience, conflict, and spiritual coping (Okan & Ekşi, 2025; Okan & Şahin, 2024; Okan et al., 2024), the lack of a tool that originally measures spiritual amnesia draws attention.

This study aims to develop a scale that can systematically measure spiritual amnesia and seeks to answer the following basic question: “How can spiritual amnesia be measured among Turkish youth and which psychological/cultural factors are associated with it?”.

### Psychological and Social Effects of Spiritual Amnesia

It has been shown that the weakening of the spiritual bond has effects on the individual’s mental health and social relationships. Spiritual amnesia interrupts the individual’s efforts to give meaning to his/her life and increases the level of existential anxiety (Park & Edmondson, 2012). This may lead to an increase in psychological symptoms such as depression, anxiety, and stress (Koenig, 2012; Okan & Şahin, 2024; Pargament et al., 2013a, 2013b). Digital amnesia, especially in young individuals, weakens not only information access habits but also mental representations of symbols and values associated with spirituality (Kanbay et al., 2025; Robert et al., 2024). Pushing spiritual contents to the background in the fast-consuming digital information flow can lead to distraction, blurring of meaning, and spiritual memory gaps in the individual.

Spiritual amnesia also has negative effects on the individual’s identity development. Especially in adolescence and young adulthood, the relationship that an individual establishes with spiritual values is critical for identity formation (Smith & Snell, 2009). Spiritual disconnections experienced in this period may cause inconsistencies, ambivalence, and identity confusion in the individual’s self-perception.

At the social level, spirituality acts as a bridge for the individual to establish a bond with the social structure to which he/she belongs. In collectivist societies like Turkey, religious and spiritual values strengthen the harmony of the individual with the social environment (Aygün & İmamoğlu, 2002). The weakening of these ties may lead to negative consequences such as loneliness, social exclusion, and lack of social support (Yılmaz & Göksu, 2021; Zhang & Andl, 2022). Individuals experiencing spiritual amnesia may experience rapid individualisation, distancing from social norms, alienation from traditional value systems, and weakening of the sense of belonging (Anderson & Subbulakshmi, 2024).

### Scale Development Studies on Spiritual Amnesia

The number of scales that directly measure spiritual forgetfulness or religious disconnection is limited in the literature. Some existing scales are as follows:

- *Spiritual Well-Being Scale (SWBS)*: Assesses spiritual well-being (Paloutzian & Ellison, 1982).
- *Spiritual Struggles Scale*: Measures spiritual conflicts and crises (Exline et al., 2014).
- *Faith Maturity Scale*: Examines the effect of faith on the individual (Benson et al., 1993).

The contribution of this study is the development of a conceptually grounded scale that specifically measures spiritual forgetfulness and is appropriate for the Turkish cultural context.

### Purpose of the Study

In this context, the main objectives of the study are as follows:

1. To reveal the conceptual framework of spiritual amnesia,
2. To analyse the psychological and social effects of this phenomenon,
3. To develop a valid and reliable scale to measure spiritual amnesia.

The results of this study are expected to contribute both to the spiritual psychology literature and to the fields of applied psychological counselling and social policy.

## Method

### Design and Purpose of the Study

This research is a quantitative study based on scale development approach. The main purpose of the research is to develop a valid and reliable measurement tool that assesses individuals’ tendencies towards spiritual values, religious beliefs, and spiritual practices such as apathy, forgetfulness, or disconnection. Accordingly, the research process consists of three main stages:

1. Item development based on the theoretical framework and exploratory factor analysis (EFA) (Fabrigar et al., 1999; Tabachnick & Fidell, 2019),
2. Confirmatory factor analysis (CFA) to validate the model (Brown, 2015; Kline, 2016),
3. Testing the psychometric properties of the scale with validity and reliability analyses (Hair et al., 2020; Nunnally & Bernstein, 1994).

### Participants and Measurement Tools Used

The research was conducted with a total of 592 participants consisting of three different data sets. In the first stage, data were collected from 296 individuals (198 females, 98 males) for EFA; in the second stage, data were collected from 242 individuals (155 females, 87 males) for CFA; and in the last stage, data were collected from 54 individuals (39 females, 15 males) for criterion validity. In accordance with established psychometric guidelines, data for the exploratory factor analysis (EFA) and confirmatory factor analysis (CFA) were collected from two separate participant groups at different time points. A total of 296 subjects were recruited for EFA in the initial data collection phase, while a distinct sample of 242 subjects participated in the CFA during a subsequent phase. The rationale behind this separation was twofold: firstly, to circumvent the issue of overfitting, and secondly, to ensure that the factorial structure identified in EFA could be independently validated. All of the participants consisted of individuals in emerging adulthood between the ages of 18 and 35. Demographic information form and scale form were applied to the participants, and convenience sampling method was preferred in sample selection. Detailed demographic information is presented in Table 1.

**Table 1:** Participant information

Age group	EFA women	EFA male	EFA total	CFA female	CFA male	CFA total	Criterion validity female	Criterion validity male	Criterion validity total
17–20	89	46	135	72	34	106	13	5	18
21–25	91	45	136	68	38	106	16	9	25
26–35	18	7	25	15	15	30	10	1	11
Total	198	98	296	155	87	242	39	15	54

### Scale Development Process

Table 2 provides a general overview of the scale development process.

**Table 2:** Steps in the development of the spiritual amnesia scale (Field, 2018; Koenig & Al Zaben, 2021)

Stage	Process title	Description	Key references
1	Theoretical framework establishment	The concept of spiritual amnesia was defined as the weakening of cognitive, emotional, and behavioural connections to spiritual values	Pargament (1997); Hill and Pargament (2003); Koenig (2012); Koenig and Al Zaben (2021)
2	Item pool generation	Items were developed based on spiritual disengagement, value-memory loss, and inconsistency in faith-based behaviour	Fetzer Institute (1999); Koenig et al. (2001); Connor and Davidson (2003); Koenig and Al Zaben (2021)
3	Expert review	Feedback was collected from 5 experts in spiritual psychology, counselling, and assessment. Scale contamination and construct overlap were considered	Clark and Watson (1995); DeVellis (2016); Bambling (2025); Koenig and Carey (2024)
4	Pilot testing	A small sample (*n* = 39) was used to test the clarity, readability, and cultural appropriateness of the items	DeVellis (2016); Hinkin (1998); Wagnild and Young (1993)
5	Exploratory factor analysis (EFA)	Factor structure and potential subdimensions were analysed. Items with low loadings were eliminated	Costello and Osborne (2005); Fabrigar et al. (1999)
6	Confirmatory factor analysis (CFA)	The structure identified via EFA was tested using model fit indices to confirm dimensionality	Byrne (2016); Hu and Bentler (1999); Schreiber et al. (2006)
7	Criterion validity	The SAS was correlated with the Spiritual Contradiction Scale. Scale contamination and overlapping constructs were taken into account	Cronbach & Meehl (1955); Koenig and Carey (2024); Koenig and Carey (2025)

#### Theoretical Base

The Spiritual Amnesia Scale (SAS) aims to measure the weakening of individuals’ mental, emotional, and behavioural ties to spiritual values over time. Spiritual amnesia is defined as the weakening of the cognitive connection with beliefs, loss of meaning, and distancing from spiritual practices; this situation can negatively affect the individual’s psychological resilience and capacity to produce meaning (Frankl, 1963; Koenig, 2012; Pargament, 1997). Sociocultural transformations such as digitalisation and individualisation accelerate this process, and contemporary concepts such as digital amnesia make it difficult to keep spiritual content at the mental level (Ekşi et al., 2021; Kanbay et al., 2025; Zhang & Andl, 2022). SAS contributes to measuring the psychological adaptation of individuals by evaluating their spiritual forgetfulness levels in this process.

#### Creating an Item Pool

Scale items were created based on literature review and expert opinions on spirituality psychology, digitalisation, and forgetting. Initially, a pool of 27 items was created and reduced to 23 items after expert evaluations. The items include dimensions such as individuals’ mental indifference to spiritual values, decrease in religious practices, and loss of meaning. With the pilot application results and EFA-DFA analyses, the scale was finally reduced to 19 items.

#### Expert Evaluation

The scale items were evaluated for content validity by five academicians who are experts in the field of spirituality and psychological counselling. Feedback was received within the framework of conceptual coherence, linguistic clarity, and suitability for the target age group, and linguistic and contextual revisions were made to eliminate ambiguity in some items. This process strengthened the conceptual integrity of the SAS.

#### Pilot Application

The scale was piloted on 39 participants between the ages of 17 and 35; the comprehensibility of the items, clarity of the instructions, and cultural appropriateness were evaluated. Small-scale wording corrections were made in line with the feedback received from the participants. This step improved the appropriateness of the scale for the target group and the quality of the application.

The overall scale development process was aligned with the psychometric validation procedure outlined by Koenig and Al Zaben (2021), which emphasises independent samples for EFA and CFA, robust content validation through expert review, and the avoidance of conceptual contamination by ensuring that items exclusively target the intended spiritual constructs rather than overlapping with mental or social health indicators.

### Data Collection and Analysis Process

The data collection process of the study was carried out with a sample of individuals in emerging adulthood. The Spiritual Amnesia Scale (SAS) and measurement tools including demographic variables were applied to the participants; the data collection process was carried out through both face-to-face interviews and online surveys. Participation was voluntary and participant confidentiality was meticulously protected. The data were digitally coded and transferred to SPSS and AMOS programmes in order to carry out statistical analyses. In order to test the validity and reliability of the scale, a multi-stage analysis process was followed. Firstly, exploratory factor analysis (EFA) was conducted to evaluate the construct validity and items with low factor loadings were eliminated. The factor structure based on the EFA findings was then tested with confirmatory factor analysis (CFA) and the goodness-of-fit values of the model were found to be compatible with the criteria suggested in the literature. In the content validity assessment, Lawshe (1975) technique was applied to determine the suitability of each item in line with expert opinions and content validity ratios were calculated.

Within the scope of criterion validity, Pearson correlation analysis was performed between the SAS and the Spiritual Contradiction Scale, which aims to measure similar constructs, and a statistically significant and positive relationship was found between the two scales (*r* = 0.448, *p* < 0.001). This finding shows that the SAS is at a sufficient level in terms of external validity. Within the framework of reliability analyses, the internal consistency of the scale was tested with Cronbach’s Alpha coefficient and a high level of reliability (*α* = 0.964) was obtained. In addition, item-total correlation coefficients were analysed; all items showed values above 0.60, which revealed that the scale had a homogeneous structure. These findings strongly support that the SAS is a valid and reliable psychometric tool for measuring the level of spiritual amnesia.

## Findings

### Findings Related to Scale Development

In this section, the development process of the Spiritual Amnesia Scale (SAS) and the statistical findings obtained are discussed in detail. The steps followed during the development of the scale and the findings obtained in this process are presented in a structured manner.

#### Validity

The validity and reliability of a scale are one of the main factors determining its suitability for use in scientific studies. In this study, the validity of the Spiritual Amnesia Scale was evaluated through various analyses. Validity is related to the ability of the scale to measure the targeted concept in a purposeful and accurate manner. As a result of the validity analyses of this scale developed to evaluate the concept of spiritual amnesia, findings supporting the construct validity of the scale and its suitability for the purpose of measurement were obtained. In this context, in order to evaluate the validity of the scale, the factor structure was determined by exploratory factor analysis (EFA) and the accuracy of this structure was tested by confirmatory factor analysis (CFA). In addition, content validity based on expert opinions and criterion validity through correlation analyses with other valid scales were evaluated.

#### Exploratory Factor Analysis (EFA) Findings

The results of exploratory factor analysis (EFA) show that the Spiritual Amnesia Scale has a unidimensional structure. The first component (factor) explained 61.173% of the total variance, and this revealed that the scale had sufficient construct validity. According to the social sciences literature, a total variance explained between 40 and 60% is considered sufficient (Okan & Okan, 2024; Hair et al., 2020; Tabachnick & Fidell, 2019).

The single-factor structure in Table 3 supports that the concept of spiritual amnesia is measured in a holistic structure representing the harmony between individuals’ spiritual values, beliefs, and behaviours. According to the factor analysis results, the Spiritual Amnesia Scale showed that the behaviours and attitudes associated with spiritual amnesia could be assessed in accordance with the theoretical basis. These findings confirm that the scale has a robust construct validity and is a reliable measurement tool that can be used in scientific research.

**Table 3:** Variance explained for spiritual amnesia scale as a result of EFA

Factor	Total explained variance
Spiritual amnesia	% 61.173
Total	% 61.173

According to Table 4, Kaiser–Meyer–Olkin (KMO) Sampling Adequacy value was calculated as 0.961. The KMO test is a criterion that evaluates the suitability of the data set for factor analysis and according to the generally accepted criteria (0.90 and above is excellent; 0.80–0.90 is very good; 0.70–0.80 is moderate; 0.60–0.70 is poor; below 0.60 is inadequate). In this context, the value of 0.961 indicates that the data set is highly suitable for factor analysis and provides a strong basis for the construct validity of the Spiritual Amnesia Scale. Bartlett’s Test of Sphericity results also confirm the suitability of the data set for factor analysis. The Chi-square value was 4739.062, the degrees of freedom (df) was 171, and the significance level was *p* < 0.000. This test indicates that there are significant correlations between the scale items and the data can be analysed in terms of multivariate factor structure. These results clearly demonstrate that the Spiritual Amnesia Scale has sufficient sample size and appropriate data characteristics to determine the factor structure. In the light of these findings, it can be concluded that the validity studies of the scale with factor analysis can be carried out reliably.

**Table 4:** KMO and Bartlett’s test values

Kaiser–Meyer–Olkin sampling adequacy		0.961
Bartlett’s test of sphericity	Chi-square value	4738,062
	S. degree	171
	*p*	0.000

**Fig. 1:**
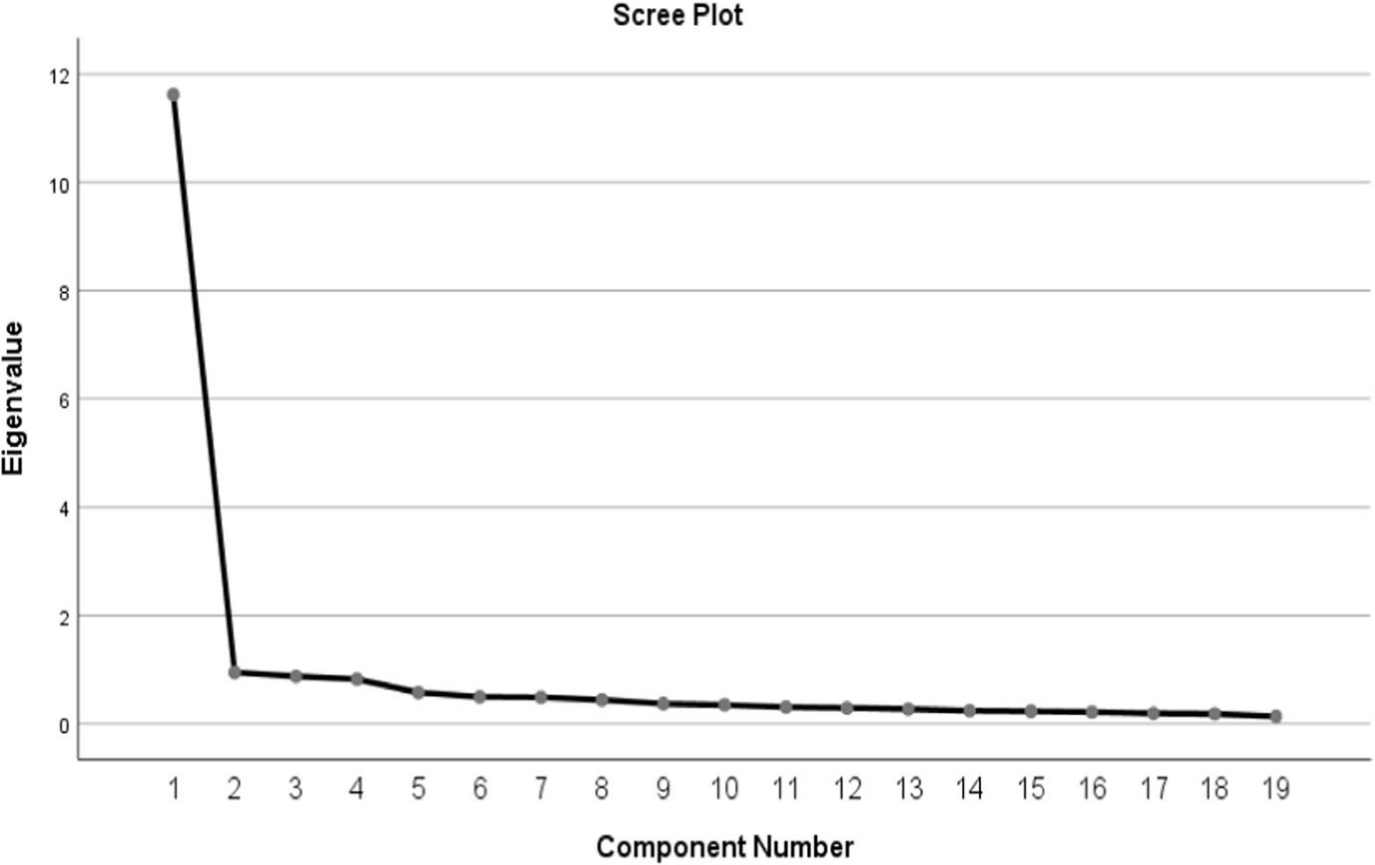
Scree plot graph for the scale

Scree Plot Fig. 1 is a tool used to assess the factor structure of the scale and provides a visual analysis based on the eigenvalues of the factors. When evaluated for the Spiritual Amnesia Scale, there is an elbow point clearly visible in the graph. In the graph, a significant decrease in slope is observed after the 1st factor and the slope flattens after this point. This shows that the scale has a single-factor structure. The first factor explains most of the scale with the highest eigenvalue. The eigenvalue of the 1st factor is above 1 and represents a significant part of the variance in the scale. Since the eigenvalues of the components following this factor are below 1, it is considered that these components do not make a significant contribution to the structure of the scale. The first factor explains a large portion of the total variance of the scale. This slope change, which is clearly seen in the Scree Plot graph, confirms that the Spiritual Amnesia Scale is a unidimensional scale. The low eigenvalues of the other factors support the unidimensional structure of the scale, which captures the overarching construct of spiritual amnesia.

Table 5 presents the factor loadings of the Spiritual Amnesia Scale (SAS) and strongly supports the unidimensional structure of the scale. The factor loading of each item reflects the level of representation of the item in question to the concept of spiritual amnesia. The factor loadings in the table vary between 0.565 and 0.867, and these values indicate that the scale items overlap with the theoretical structure at a high level.

**Table 5:** Load values of spiritual amnesia scale items

Articles	Spiritual amnesia
Item16	0.867
Item7	0.831
Item15	0.828
Item9	0.825
Item10	0.820
Item17	0.818
Item13	0.817
Item6	0.807
Item12	0.806
Item11	0.804
Item18	0.803
Item8	0.790
Item5	0.776
Item19	0.768
Item14	0.736
Item3	0.735
Item4	0.730
Item2	0.682
Item1	0.565

According to the psychometric literature, factor loadings of 0.40 and above are acceptable and indicate that the scale items are sufficient to represent the relevant construct (Hair et al., 2020; Tabachnick & Fidell, 2019). In this context, SAS items, with their high factor loadings, effectively reflect the tendencies of individuals to forget, neglect, or develop indifference towards their ties with religious and spiritual values.

The fact that the items have high and close loadings indicates that the scale is constructed in conceptual integrity and can successfully evaluate the concept of spiritual amnesia under a single holistic structure. This situation reveals that SAS is a powerful tool in measuring the tendency of individuals to distance themselves from spiritual beliefs, values, and practices. The findings indicate that the SAS has high construct validity and is a valid and reliable measurement tool that can be used especially in the empirical evaluation of religious/spiritual amnesia processes.

#### Confirmatory Factor Analysis

**Fig. 2:**
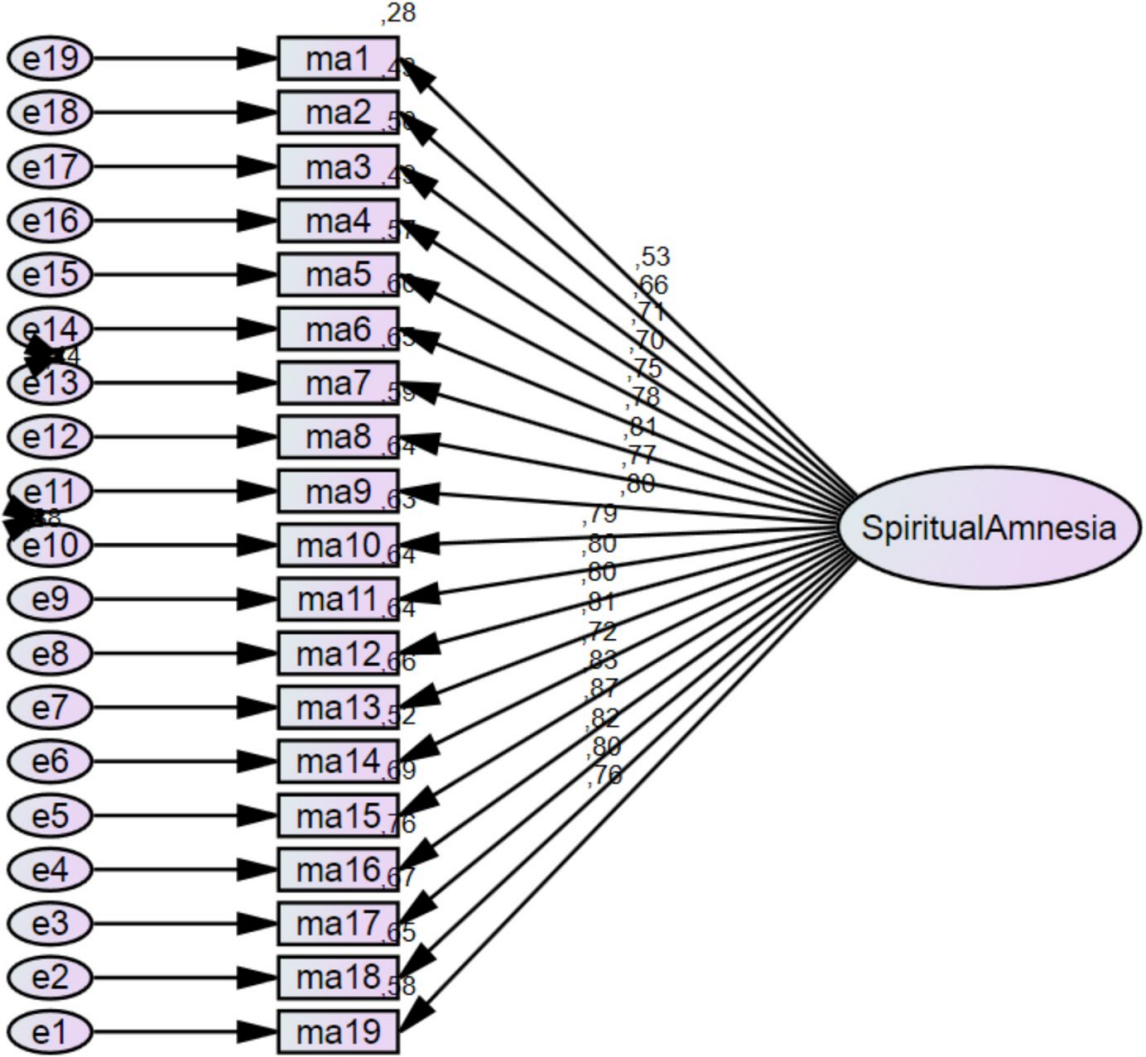
Confirmatory factor analysis path diagram of spiritual amnesia scale

The CFA diagram in Fig. 2 reflects the details of the confirmatory factor analysis model for the Spiritual Amnesia Scale. The diagram reveals the unidimensional structure of the scale and the relationships between the items and this dimension. A detailed analysis of this diagram is given below: Factor loadings represent the relationship between the items and the Spiritual Amnesia dimension. The factor loadings in the figure range between 0.53 and 0.87. Item15 (0.87): This item stands out as the item that most strongly represents the conceptual structure of the scale. Item14 (0.83): It is another strong representative of the scale and makes a high contribution to the Spiritual Amnesia dimension. Item1 (0.53 and Item2 (0.66): These items support the overall structure of the scale with an acceptable loading value. The scale was modelled with a single-factor, Spiritual Amnesia dimension. The majority of the items had loading values of 0.70 and above and consistently represented the concept of Spiritual Amnesia. The loading values of all items were 0.53 and above, indicating a strong fit with the overall scale and that all items made significant contributions to the measurement. The fact that factor loadings were generally high (especially 0.70 and above) supports the unidimensional structure of the model and shows that the scale is compatible with the conceptual framework. Even items such as Item1 and Item2, which have the lowest loading values, contribute to the overall structure of the scale with acceptable loading values. This confirmatory factor analysis strongly supports that the unidimensional structure of the Spiritual Amnesia Scale is valid and that the scale provides a measurement in accordance with the conceptual framework. The loading values of all items were 0.53 and above, indicating that the items of the scale are effective in representing the Spiritual Amnesia dimension. These results reveal that the scale is a valid and reliable measurement tool that can be used in scientific studies. The overall structure of the scale is consistent and strong.

Table 6 presents the goodness-of-fit values of the confirmatory factor analysis (CFA) conducted for the Spiritual Amnesia Scale and the comparison of these values with standard criteria. According to the results of the analysis, the goodness-of-fit values of the model are generally acceptable or good fit. The c^2^/df value was found to be 2.812, and this value revealed that it was a model with an acceptable fit in the range of 2 ≤ c^2^/df ≤ 3. RMSEA value was calculated as 0.08, and this value indicates an acceptable fit. SRMR value is in the range of 0.054 and 0.05 ≤ SRMR ≤ 0.10 and shows that the model provides an acceptable fit with the data. IFI (0.911) and CFI (0.911) values are between 0.90 ≤ fit criteria ≤ 0.95 and TLI (0.897) value is in the range of 0.85 ≤ fit criteria ≤ 0.90, reflecting the acceptable fit level. All these values show that the unidimensional structure of the model is valid and the scale works consistently with the data. While an acceptable fit in basic criteria such as RMSEA and SRMR supports that the overall fit of the model is quite strong, additional goodness-of-fit indices such as CFI, IFI, and TLI confirm that the scale is construct valid and designed in accordance with the theoretical framework. These results reveal that the Spiritual Amnesia Scale is a valid and reliable measurement tool that can be used safely in scientific research.

**Table 6:** Comparison of standard goodness-of-fit criteria and research results

Fit dimensions	Good fit	Acceptable compliance	Concordance values obtained in the study
c2/df	0 ≤ c2/df ≤ 2	2 ≤ c2/df ≤ 3	2.812
RMSEA	0 ≤ RMSEA ≤ 0.05	0.05 ≤ RMSEA ≤ 0.08	0.08
SRMR	0 ≤ SRMR ≤ 0.05	0.05 ≤ SRMR ≤ 0.10	0.054
IFI	0.95 ≤ NFI ≤ 1.00	0.90 ≤ NFI ≤ 0.95	0.911
CFI	0.95 ≤ CFI ≤ 1.00	0.90 ≤ CFI ≤ 0.95	0.911
TLI	0.90 < RFI < 1.00	0.85 < RFI < 0.90	0.897

### Reliability Findings

#### Criterion Validity of the Scale

In order to determine the criterion validity of Spiritual Amnesia Scale, Spiritual Contradiction Scale developed by Okan et al. (2024) was applied to 54 individuals in emerging adulthood. The reason for using Spiritual Contradiction Scale is that Spiritual Amnesia and Spiritual Contradiction have some common situations in the literature. The data related to this are presented in Table 7.

**Table 7:** Relationship between spiritual amnesia and spiritual contradiction scale

Variables	1	2
Spiritual amnesia	1.00	0.448*
Spiritual contradiction		1.00

^*^p < 0.001

In order to evaluate the criterion validity of the Spiritual Amnesia Scale (SAS), the Spiritual Contradiction Scale is developed by Okan et al. The main reason for examining the relationship between these two scales is that both scales are intended to assess the level of incompatibility between individuals’ spiritual values, beliefs, and behaviours. Within the scope of the study, both scales were applied to 54 participants in emerging adulthood. As a result of the analysis, a significant and positive correlation was found between Spiritual Amnesia and Spiritual Contradiction scores (*r* = 0.448, *p* < 0.001). This finding indicates that as the level of spiritual disconnection increases, the experience of moral and belief-based inner contradiction may also increase in individuals. The fact that spiritual amnesia results in loss of meaning in the individual’s value system and distancing from beliefs may increase moral inconsistencies and uncertainties in ethical decision-making processes. Therefore, this relationship supports the criterion validity of the SAS and also emphasises the effect of spiritual values on the moral and behavioural consistency of the individual. The findings suggest that the SAS is a valid and functional tool for assessing individuals’ levels of spiritual disconnection and relating it to broader ethical-spiritual processes such as Spiritual Contradiction.

Table 8 shows the unidimensional structure of the Spiritual Amnesia Scale (SAS), the item-total correlations for each item, and the “Cronbach’s Alpha” values obtained when the item was removed. The overall Cronbach’s Alpha coefficient of the scale was calculated as 0.964, which indicates that the scale has an extremely high internal consistency and can reliably measure the concept of spiritual amnesia.

**Table 8:** Internal consistency coefficients of spiritual amnesia scale sub-dimensions and item-total correlations and Cronbach’s alpha values

Articles	Corrected item-total correlation	Cronbach’s alpha if item deleted
Spiritual Amnesia		0.964
Item1	0.532	0.965
Item2	0.649	0.963
Item3	0.705	0.963
Item4	0.703	0.963
Item5	0.747	0.962
Item6	0.779	0.962
Item7	0.805	0.961
Item8	0.761	0.962
Item9	0.800	0.962
Item10	0.795	0.962
Item11	0.775	0.962
Item12	0.778	0.962
Item13	0.789	0.962
Item14	0.704	0.963
Item15	0.801	0.962
Item16	0.845	0.961
Item17	0.793	0.962
Item18	0.773	0.962
Item19	0.737	0.962

Item-total correlation values ranged between 0.532 and 0.845. In the social sciences literature, correlation values above 0.30 indicate that each item contributes significantly to the overall structure of the scale (Nunnally & Bernstein, 1994). Accordingly, it can be said that all items support the conceptual integrity of the scale. Especially items such as Item16 (0.845) and Item7 (0.805) have stronger discrimination in terms of assessing the level of spiritual amnesia and can reliably reflect the level of weakness in individuals’ relationships with spiritual values.

There was no significant decrease in Cronbach’s Alpha value when each item was removed. This shows that the structural integrity of the scale is not dependent on any single item, and that all items work together to represent a homogenous structure.

These findings statistically confirm the theoretically predicted unidimensional structure of the Spiritual Amnesia Scale and reveal that the individual can evaluate the relationship with spiritual values, the weakening in this relationship, and the tendency to forget in a holistic structure. The scale is suitable to be used in academic researches as a valid and reliable tool for measuring spiritual amnesia at the individual level.

According to the test–retest analysis presented in Table 9, the mean total scores of the Spiritual Amnesia Scale in the first and second administrations were 56.32 (± 13.21) and 56.87 (± 13.05), respectively. The mean difference was only 0.55 points, which was not statistically significant; however, the test–retest correlation coefficient (*r* = 0.76, *p* < 0.001) revealed that the scale showed a high level of consistency over time. This finding suggests that the scale is a reliable measurement tool and can consistently assess individuals’ levels of spiritual amnesia.

**Table 9:** Test–retest analysis results

Application	1st application (Mean ± SD)	2nd application (Mean ± SD)	Difference (ΔOrt)	Correlation (*r*)	Significance (*p*)
Total points	56.32 ± 13.21	56.87 ± 13.05	− 0.55	0.76	*p* < .001

## Conclusion, Discussion, and Recommendations

### General Evaluation of Results and Findings

In this study, Spiritual Amnesia Scale (SAS) was developed and its psychometric properties were evaluated. The results of exploratory factor analysis (EFA) revealed that the scale has a single-factor structure. The scale explained 61.173% of the total variance, indicating that it has a strong construct validity within the validity limits accepted in the social sciences literature (Hair et al., 2020; Tabachnick & Fidell, 2019). The Kaiser–Meyer–Olkin (KMO) value was calculated as 0.961 and Bartlett’s Test was significant (*χ*^2^(171) = 4738.062, *p* < 0.000), which confirms that the scale is highly suitable for factor analysis. Confirmatory factor analysis (CFA) results showed that the scale had acceptable and good fit indices in terms of model fit. CFI = 0.911, IFI = 0.911, and RMSEA = 0.08 values reveal that the one-factor model is consistent with the theoretical framework (Hu & Bentler, 1999). The Cronbach’s Alpha value calculated within the scope of reliability analysis was 0.964, indicating that the scale has high internal consistency (Nunnally & Bernstein, 1994).

Within the scope of the criterion validity of the scale, a positive correlation (*r* = 0.448, *p* < 0.001) was found with the Spiritual Contradiction Scale (Okan et al., 2024). The correlation coefficient obtained in the test–retest analysis was calculated as 0.76 (*p* < 0.001), indicating that the scale provides stable measurement over time. All these findings indicate that the Spiritual Amnesia Scale is a psychometrically valid and reliable measurement tool.

### Discussion

The objective of this study was to develop an original scale with which to systematically measure the phenomenon of spiritual amnesia, defined as the loss of religious and spiritual ties over time. In addition, the study evaluated the psychometric properties of the Turkish Spiritual Amnesia Scale (TSAS). Despite the existence of scales that address concepts such as spiritual well-being (Paloutzian & Ellison, 1982), spiritual struggles (Exline et al., 2014), and faith maturity (Benson et al., 1993), there is a conspicuous absence of instruments that tackle the progressive disengagement from spiritual identity—what is termed “spiritual amnesia”—with conceptual integrity and psychometric rigour. Spiritual amnesia is a multifaceted process characterised by the dissolution of the connection to spirituality across the domains of cognition, emotion and behaviour (Hill & Pargament, 2003; Pargament, 1997). As postulated by Park and Edmondson (2012), Koenig (2012) and Pargament et al., (2013a, 2013b), the phenomenon is associated with disruptions in meaning-making, reduced engagement in rituals, and emotional alienation from transcendent values. The present study’s findings align with this framework, demonstrating that spiritual amnesia is experienced holistically, as reflected in the unidimensional yet theoretically rich structure of the TSAS. The present study makes a theoretical contribution through the introduction of a novel operationalisation of spiritual detachment, thereby advancing extant theories of spirituality by means of the empirical capture of weakening spiritual ties. The TSAS thus establishes a connection between conceptual work and measurable dimensions, thereby creating new pathways for both theoretical elaboration and practical assessment.

The role of digitalisation and contemporary lifestyles in shaping spiritual detachment has received increasing attention from scholars (Anderson & Subbulakshmi, 2024; Kanbay et al., 2025; Robert et al., 2024). The notion of “digital amnesia” encapsulates the cognitive and emotional overload experienced by digitally native youth, who may develop fragmented or superficial spiritual routines. This finding is consistent with observations that memory, spirituality, and moral anchoring are increasingly influenced by algorithmic cultures and instant-access environments (Natale et al., 2024; Zhang & Andl, 2022). In the case of Turkish adolescents, for instance, the substitution of communal prayer traditions with individual mobile applications can result in a sense of disconnection, despite the technological facilitation of faith.

Within collectivist societies such as Turkey, spiritual amnesia also bears social costs. The weakening of ties with religious identity has been demonstrated to result in increased individualism, loss of belonging, and psychosocial vulnerability (Aygün & İmamoğlu, 2002; Smith & Snell, 2009; Yılmaz & Göksu, 2021). Recent findings also suggest that spiritual orientation indirectly affects mental health outcomes through its influence on meaning-making and death-related attitudes (Söylev et al., 2025). This supports the current study’s interpretation of spiritual amnesia as a psychologically consequential construct. In particular, young adults may experience identity confusion and social isolation when attempting to reconcile inherited religious norms with modern cultural shifts. In this sense, the TSAS is not only relevant for individual assessment but also for community-based interventions in education, counselling, and youth policy.

The scale’s criterion validity, supported by a significant correlation with the Spiritual Contradiction Scale (*r* = 0.448, *p* < 0.001), suggests that spiritual amnesia is closely linked with internal struggles around ethics, morality, and values (Ahmadi & Ahmadi, 1998). For instance, Turkish youth may persist in identifying with Islamic values whilst simultaneously reporting a disengagement from ritualistic practices. This incongruity serves as an exemplification of the phenomenon the TSAS aims to quantify. Consequently, the scale demonstrates considerable potential for application in the domains of moral psychology, religious education, and value-based counselling. It is important to note that the TSAS may serve practical functions for spiritual counsellors, school psychologists, and youth mental health professionals who aim to assess spiritual disconnection not as a deficit, but as a context-bound and reversible process. The scale provides a culturally grounded tool for detecting early signs of existential distress and value disengagement.

In conclusion, this study has provided a new psychometric instrument and a conceptual contribution to understanding spiritual disconnection at psychological, social, and cultural levels. The TSAS has been demonstrated to possess robust indices of validity and reliability, thus rendering it a promising instrument for both academic research and applied practice. It is recommended that subsequent studies examine the scale in culturally distinct contexts, including Muslim diaspora communities in Europe, secularised youth populations in East Asia, and post-conflict regions in the Middle East, where spiritual detachment may manifest in unique forms. Cross-cultural validation will enhance the tool’s generalisability and contribute to a deeper understanding of contemporary spirituality under global transformation.

### Limitations and Suggestions for Future Studies

Although the results of this study make an important contribution to the development of a valid and reliable scale to measure spiritual amnesia, several limitations should be acknowledged.

#### Limitations

First, the data were collected from a sample restricted to a specific cultural and religious context (i.e. Turkish youth), which may limit the generalisability of the findings. Secondly, the cross-sectional design of the study does not allow for an examination of the temporal stability or long-term psychological effects of spiritual amnesia. In addition, self-report measures may be at risk of social desirability bias or introspective limitations, particularly for constructs involving spirituality or religiosity. Finally, although initial indicators of validity and reliability are promising, further validation with clinical samples and diverse populations is needed.

#### Suggestions for Future Research

In this context, the following suggestions may serve as guidelines for future research:

- The application of the scale in different cultural groups: In order to comprehend the manifestation of spiritual amnesia across sociocultural contexts, it is imperative that the scale be tested in other countries and cultures.
- The scale’s correlation with long-term psychological effects: Longitudinal studies can be conducted to explore the long-term impacts of spiritual amnesia on individuals’ mental health.
- The relationship with psychological resilience and search for meaning: Future research could examine the relationship between spiritual amnesia and individuals’ levels of psychological resilience and sense of meaning in life.
- The scale’s application in spiritual counselling practices: The scale may be utilised in clinical settings to assess how individuals experience spiritual disconnection within spiritual counselling processes.
- Connection with secularisation and individualisation processes: Sociological investigations could explore how processes such as digitalisation and modernisation contribute to the emergence or increase in spiritual amnesia.

In line with these suggestions, the potential use of the Spiritual Amnesia Scale in various domains may be enhanced, allowing for a more comprehensive understanding of its implications on individual well-being.

### Conclusion

The present study sets out to develop and evaluate a novel instrument for measuring spiritual amnesia, defined as the progressive weakening of individuals’ ties with religious and spiritual values over time. The Turkish Spiritual Amnesia Scale (TSAS) delineates the conceptual boundaries of this under-explored phenomenon, offering an objective and theoretically grounded means of assessing spiritual disconnection among young individuals in the Turkish context. The findings demonstrate that the TSAS has high construct validity and internal consistency. Exploratory and confirmatory factor analyses support a unidimensional structure, suggesting that tendencies such as forgetting, neglecting, and emotionally disconnecting from spiritual content can be meaningfully captured as a unified psychological construct. The test–retest analysis further confirmed the scale’s temporal reliability. It is important to note that the TSAS is not only psychometrically robust but also functionally versatile. The study provides insight into key psychosocial dimensions, including the loss of existential meaning, detachment from rituals, diminished spiritual motivation, and reduced sense of social belonging. It is evident that these dimensions frequently manifest in response to macro-level cultural shifts, encompassing modernisation, digitalisation, and individualisation. From an applied perspective, the TSAS holds practical value for professionals in spiritual counselling, youth guidance, and clinical psychology. It has the capacity to function as both a diagnostic instrument and a monitoring tool for the identification of spiritual struggles in adolescence and young adulthood. Furthermore, it may provide a foundation for the integration of interventions within the domains of moral education, character development programmes, and religious education curricula. It is recommended that future research be conducted to validate the TSAS in a broader population. The suggested directions for further research include Muslim diaspora communities in Europe, youth groups in highly secularised societies such as Scandinavia or Japan, and transitional cultural contexts such as post-Soviet Central Asia. Cross-cultural validation will facilitate a more profound comprehension of spiritual detachment in diverse sociocultural contexts, thereby enhancing the scale’s generalisability and international relevance. In summary, the present study makes a theoretical innovation and empirical validation of a tool in the fields of spiritual psychology, cultural psychology, and counselling. The TSAS has the capacity to assist scholars and practitioners in comprehending and addressing the dynamics of spiritual disconnection in a rapidly transforming world.

## Data Availability

The datasets generated during and/or analysed during the current study are available from the corresponding author on reasonable request.
